# Work situation of rheumatologists and residents in times of COVID-19: Findings from a survey in Germany

**DOI:** 10.1007/s00393-021-01081-5

**Published:** 2021-09-17

**Authors:** Ellen Kuhlmann, Luzia Bruns, Kirsten Hoeper, Marianne Richter, Torsten Witte, Diana Ernst, Alexandra Jablonka

**Affiliations:** 1 Klinik für Rheumatologie und Immunologie, Medizinische Hochschule Hannover, OE 6830, Carl-Neuberg-Str. 1, 30625 Hannover, Germany; 2 Regionales Kooperatives Rheumazentrum Niedersachsen e. V., Hannover , Germany

**Keywords:** Rheumatology, Stress, Task delegation, COVID-19, Germany, Rheumatologie, Stress, Aufgabendelegation, COVID-19, Deutschland

## Abstract

**Background and objective:**

The work situation is an important dimension of professional life and wellbeing, and a policy lever to strengthen recruitment and retention. This study aims to explore the work situation of physicians and residents in internal medical rheumatology, considering the impact of the coronavirus pandemic COVID-19.

**Methods:**

A questionnaire-based online survey was conducted in early 2021 at the Hannover Medical School, supported by the German Society of Rheumatology. Target groups were all rheumatology physicians and residents in Germany. The main areas of investigation included work hours, task delegation, and collaboration; workload and mental health issues; discrimination and sexual harassment experiences; and the impact of COVID-19. Descriptive statistical analysis was performed for the standardized items and qualitative content analysis for the free-text information.

**Results:**

The respondents (*n* = 101) expressed positive attitudes towards cooperation and task delegation to medical assistants, especially those specialized in rheumatology, while attitudes towards cooperation with GPs pointed to blockades. There was a strong mismatch between actual and desired work hours both in the group of women and in the group of men. 81% rated their workload as high or very high; every sixth rheumatologist has suffered from stress or burnout syndromes at least once in the past. Experiences of gender discrimination and sexual harassment/violence were frequently reported, mostly by women. COVID-19 was an amplifier of stress, with major stressors being digitalization and increased demand for communication and patient education.

**Conclusion:**

There is an urgent need to improve the work situation of rheumatologists and reduce stress and mental health risks.

**Supplementary Information:**

The online version of this article (10.1007/s00393-021-01081-5) includes the survey questionnaire in German.

## Abstract (de)

**Hintergrund und Fragestellung:**

Die Arbeitssituation ist ein wichtiger Aspekt im Berufsleben und für das Wohlergehen und ein politischer Hebel, um den Verbleib im Beruf sowie die Fachkräfterekrutierung zu verbessern. Ziel dieser Studie war es, die Arbeitsbedingungen internistischer Rheumatolog*innen und Weiterbildungsassistent*innen unter Berücksichtigung der Auswirkungen der Coronavirus-Pandemie COVID-19 zu untersuchen.

**Methode:**

Eine fragebogenbasierte Online-Erhebung wurde Anfang 2021 an der Medizinischen Hochschule Hannover durchgeführt, unterstützt von der Deutschen Gesellschaft für Rheumatologie. Zielgruppe waren alle Rheumatolog*innen und Weiterbildungsassistent*innen in Deutschland. Thema waren Arbeitszeiten, Kooperation und Delegation, Arbeitsbelastungen und Burnout-Syndrome, Diskriminierung und sexuelle Belästigung sowie Auswirkungen von COVID-19. Standardisierte Items wurden deskriptiv und Freitextinformationen mittels qualitativer Inhaltsanalyse ausgewertet.

**Ergebnisse:**

In der Untersuchungsgruppe (*n* = 101) zeigten sich positive Einstellungen zu Kooperation und Aufgabendelegation an medizinische Fachangestellte, v. a. Rheumatologische Fachassistenz, während die Einstellungen zur Kooperation mit Hausärzt*innen Blockaden sichtbar machten. Die tatsächliche Arbeitszeit unterschied sich sowohl in der Gruppe der Frauen als auch der Männer sehr deutlich von der Wunscharbeitszeit. Ihre Arbeitsbelastung bewerteten 81 % als hoch oder sehr hoch; jede*r 6. Befragte war mindestens einmal in der Vergangenheit von Stress und Burnout betroffen. Diskriminierungserfahrungen und sexuelle Belästigungen waren weitverbreitet und betrafen Frauen stärker als Männer. COVID-19 erwies sich als Stressverstärker; Digitalisierung, erhöhter Bedarf an Kommunikation und Patientenaufklärung waren wesentliche Stressfaktoren.

**Schlussfolgerungen:**

Die Arbeitssituation von Rheumatolog*innen sollte dringend verbessert werden, um Stress und Gesundheitsrisiken zu verringern.

## Introduction

The work situation is an important dimension of professional life and wellbeing. Its importance goes beyond individual wellbeing and affects the profession itself, thus shaping capacity building for recruitment and retention. This is especially important in the healthcare sector, where opportunities for competition for qualified staff are generally more hampered by the politics of comprehensive regulation of education, employment, and practice conditions. The situation is even more difficult in small highly specialized medical professions, such as rheumatology, where the long duration of training and poor representation at medical schools add further challenges to recruitment [22, 27]. Rheumatology fishes from the same pool of internal medicine as other medical specialties with broadly similar professional and organizational conditions. This turns the workplace and the individual perceptions of the work situation into an arena of competition for qualified physicians.

The pressure to increase staffing levels and improve recruitment and retention is particularly strong in rheumatology [8, 21, 22]. A recent workforce assessment revealed that an available stock of physicians specialized in internal medical rheumatology cannot meet the new health policy planning targets. Health workforce trends over recent years showed the weakest increases precisely in the younger age groups; numbers even declined in the group aged 40 to 50 years [22]. In this situation, greater attention to the work situation and the perceptions, needs, and demands of the individual rheumatologists may open windows of opportunity for effective health workforce management to mitigate growing shortages.

The COVID-19 pandemic has reinforced the need for taking better care of healthcare workers and paying attention to their work situation. Disruptions of work routines and the new health threats caused by COVID-19 have significantly altered frontline work in healthcare and may even have heightened stress and workload [10, 31]. The World Health Organization reports growing evidence of fatigue in the health workforce [40], which may result in a lack of motivation, increased sickness absence and burnout, and, finally, job leaves. It is therefore crucial to understand how these conditions impact on the workplace and work life of rheumatologists.

Health workforce research [23] is poorly developed in rheumatology; microlevel and actor-centered approaches are largely absent. Thus, very little is known about rheumatologists’ individual perceptions. Below, we summarize major themes and information from a narrative literature review with a focus on three dimensions of everyday work life: the work hours and work–life balance, task delegation and cooperation, and stress and gender discrimination experiences. No information was available on the impact of the COVID-19 pandemic. Another important dimension of health workforce development in rheumatology is education and training [11, 21, 22], which, however, is not addressed in this study and is subject to further investigation.

### Working hours and work–life balance

A small empirical study carried out among resident rheumatologists in three Eastern German federal states (*n* = 27) reported a high relevance of a good work–life balance and of compatibility of work and family responsibilities; nearly half of the participants in the survey would prefer parttime work. The authors highlighted a need for alternative employment models in rheumatology to improve recruitment [17, 32]. Surveys among trainees in the United Kingdom [7] and Canada [6] also identified an appropriate work–life balance as one of the factors that may attract physicians to rheumatology. Barber et al. highlighted that work hours depend on sex category and type of practice [2]. Detailed information on work hours and workload of rheumatology physicians in Germany is not available, however.

### Task delegation and cooperation

Rearranging the allocation of tasks and skills and improving cooperation between healthcare workers and within the medical professions are key areas of innovation in the health workforce [8, 23, 29, 35]. In rheumatology, the debate is most advanced in the USA and Anglo-Saxon health systems. Nurses, especially nurse practitioners, and physician assistants have been target groups of task delegation and task shifting [14]. The “EULAR recommendations for the role of the nurse in the management of chronic inflammatory arthritis” provide an illustrative example of the tasks that may be exercised by nurses [3]. In Germany, efforts focus on task *delegation* rather than task shifting or skill mix, targeting medical assistants and those specialized in rheumatology, i.e., *Rheumatologische Fachassistenz *(RFA, specialized rheumatology assistance; Infobox 1).

#### Infobox 1 Medical assistants and specialized rheumatology assistance (RFA)

Medical assistants account for the largest group in ambulatory care in Germany; a small number may also work in hospitals, especially in outpatient services and documentation. They undergo a 3-year education in the German system of dual education (combining school-based and on-the-job training). Since 2006, specialization in ambulatory rheumatology care can be obtained (on a 60-hour curriculum) and a new 120-hour curriculum has recently been certified by the Physicians’ Chamber [5]. This is the only non-medical specialization in rheumatology in Germany; a nurse specialty program as known from other countries does not exist. Partial overlaps with the work of nurses in NHS systems and the USA might exist; some international comparative studies use the term “nurses” for specialized medical assistants. However, medical assistants are not included in the regulated professions and the European Union database [9] and no standardized international occupational category exists for this group. We use the German term “RFA” (specialized rheumatology assistance).

Research evidence and policy recommendations highlight opportunities and benefits of delegating tasks in rheumatology to medical assistants, in particular RFAs, who can provide high-quality care to patients [12, 13, 18–21, 39]. However, the debate takes a health services perspective and is concerned with quality of care and patient safety [13, 18]. The impact of task delegation and the attitudes of rheumatologists have rarely been considered from a health workforce perspective.

Another important area of workforce innovation is the cooperation between rheumatologists and general practitioners (GPs, *Hausärzte*). An Austrian study among rheumatologists and GPs investigated the therapeutic approaches and concluded that “considerable consensus between the two professional groups’ constitutes a solid base for future joint recommendations” [33]. Opportunities for cooperation were confirmed by a cross-sectional study which explored cooperation between general practitioners and several other healthcare providers in two German federal states in 2017 [36, 37]. Implementation may be challenging, as GPs perceived cooperation with rheumatologists as important, but inadequate and in need of improvement [35].

### Stress and discrimination at the workplace

There is growing awareness that “care of the patient requires care of the provider” [4]. Sensitivity for potentially harmful working conditions and knowledge about stress and burnout syndromes have also improved in rheumatology [26]. In a psychological study among rheumatology practitioners carried out in the USA in 2020 during the pandemic (*n* = 128), half of the participants reported syndromes of burnout in at least one of the surveyed domains [37]. According to another US report, 32% of the surveyed rheumatologists felt burned out, of whom 29% stated that it began after the COVID-19 pandemic [25]. A survey in the United Kingdom revealed that “[t]wo-third of responders felt anxious about the ill-effects of COVID-19 on their health and wellbeing, and one third of them were redeployed” [28]. These findings are highly alarming, but evidence for German rheumatologists is still lacking.

One important area of workplace stress is discrimination and sexual harassment/violence. In 2019, the EULAR Task Force on Gender Equity in Academic Rheumatology conducted a web-based survey among academic rheumatologists [30]. Based on 301 responses from 24 countries, the results revealed that nearly half of the respondents (48%) had experienced gender discrimination—58% of the female and 18% of the male respondents. More than every fourth respondent reported sexual harassment, but female rheumatologists were affected about three times more often than their male colleagues (31% vs. 11%) [30]. Recent research conducted in the US also revealed persisting gender disparities in academic rheumatology [27]. Specific figures for German rheumatology are not available, but results from an online survey carried out in Berlin in 2015 among all physicians working in tertiary referral centers confirmed widespread discrimination and sexual harassment in medicine in Germany. Overall, 737 participants were included in the analysis (60% women, 39% men, 1% others/unknown). About 70% reported some form of sexual harassment, most often verbal misconduct; women were affected more often than men [16]. Persisting gender inequality in academic medical centers was also reported in a European multicenter study including Germany [24].

### Aims of the study

The literature review revealed that very little is known about rheumatologists’ perceptions of their work situation, and also the impact of the COVID-19 pandemic remains a black box. The present study investigated the work situation of physicians specialized in internal medical rheumatology and residents in Germany in times of the COVID-19 pandemic (in this article, the term “rheumatologist” refers to both groups). The aim was to provide new knowledge on the work situation and the needs of rheumatologists from an actor-centered perspective of the individual physician. More specifically, major objectives were to improve individual working conditions and to strengthen workforce management in rheumatology. Four topics were addressed:

- How do rheumatologists in Germany perceive their work time and their work–life balance?
- What attitudes do rheumatologists have towards cooperation and task delegation?
- How do rheumatologists rate their levels of occupational stress and mental health risks and how are they affected by discrimination and sexual violence?
- What are the effects of the COVID-19 pandemic on the everyday work life of rheumatologists?

## Methods

We applied social sciences survey methodology and a descriptive, explorative approach. A questionnaire-based online survey was conducted at Hannover Medical School between 22 January and 28 February 2021 on the topic “health workforce, work and employment situation in rheumatology in Germany” (in German). Data were collected during the end of the second wave of the COVID-19 pandemic in Germany. The survey was supported by the German Society of Rheumatology (*Deutsche Gesellschaft für Rheumatologie e.* *V.,* DGRh); the target group comprised all rheumatology physicians and residents in Germany.

### Instrument development

The instrument development was informed by the topics identified from the literature review, amended for the COVID-19 situation. Items related to gender discrimination and sexual harassment/violence took into account the EULAR questionnaire [1, 30]. The final written questionnaire comprised seven major topics (in German; available online as Supplementary Information):

1. General information (occupational position, location).
2. Sociodemographic information (sex, age group, place of medical education).
3. Organization of work, actual and desired weekly work hours, projects supporting task delegation.
4. Attitudes towards task delegation and cooperation, opportunities to reduce shortage and impact on care quality considering GPs, medical assistants, and nurses, and RFAs.
5. Perception of workload, work–life balance, and available support structures; risks of stress and burnout and available support structures.
6. Perception of the impact of COVID-19 on the own work situation, comprising workload, delegation, and cooperation, and relevant changes at the workplace.
7. Gender discrimination, sexual harassment, and violence, and other forms of discrimination at the workplace, including own experiences and perceptions.

Another part of the questionnaire investigated the situation in resident training in rheumatology; however, this issue is not included in the present study.

The questionnaire comprised standardized items and free-text items. Items related to the occupational and organizational position were mainly explored using common categories of social and occupational statistics. Five-point Likert scales were applied to investigate perceptions and experiences, and three-point scales—yes/no/uncertain—for items related to delegation/cooperation and to discrimination. A short pre-test (*n* = 5) to assess item clarity and the time required to complete the questionnaire was carried out among the authors’ networks in the second week of January 2021. Based on the feedback, minor revisions were undertaken.

### Data collection and analysis

Ethical approval was obtained from the Ethics Committee at Hannover Medical School (November 2020; Nr 9441_BO_K_2020). The material was gathered anonymously through an established platform at Hannover Medical School in accordance with German data protection law and approved by the data protection officer at Hannover Medical School (https://tinyurl.com/Care4Rheumatology). An invitation with the survey link was distributed widely through the DGRh (https://dgrh.de) to all its members, through *Regionales Kooperatives Rheumazentrum Niedersachsen* (https://rheumazentrum-hannover.de), and through a number of other professional and personal networks of the authors.

Participants were informed about the aims and objectives of the study and written informed consent was obtained. All participants (*n* = 101) provided written consent and, with few exceptions, fully completed the main parts of the survey (on average *n* = 100; maximum 3 missing responses for a few subitems). The almost 100% completion rate indicates that the rheumatologists participating in this survey perceived the themes as relevant.

Descriptive statistical analysis was undertaken for the standardized items (SPSS, version 26, IBM Corp., Armonk, NY, USA). The decision to use an entirely descriptive analysis of the quantitative material seemed to be most appropriate given the focus on health workforce issues and the research aims and questions, as well as the research sample and the small size of subgroups—a situation which reflects more generally the conditions of small medical specialty areas. Additional free-text information was analyzed through qualitative content analysis using an inductive approach.

## Results

### The research sample

The research sample comprised *n* = 101 rheumatologists and residents reflecting various occupational, organizational, and geographical conditions. Among the respondents, younger physicians (40 years and under, *n* = 43%) and women (52%) were overrepresented. Table 1 below provides an overview of the research sample. If available, data taken from public statistics (*Gesundheitsberichterstattung*) are provided in brackets for all practicing rheumatologists (*N* = 1076; *N* = unknown for residents; for details regarding the statistical data, see [22]).

**Table 1:** The research sample

Category	Composition
Occupational group	Rheumatologists 71% (of whom 21% were authorized to train residents), residents 22%, others 7%
Sector	Ambulatory care 46% (52%)
	Hospital 41% (42%), of whom 24% were in leadership positions
Location	Eastern federal states 21%, Western federal states 79% large city 65%, middle city 27%, small city 8%
Age groups	>35 years 22% (1%), 35–40 years 21% (8%), 40–50 years 18% (25%), 50–60 years 20% (39%), 60–66 years 14% (15%), >66 years 5% (11%)
Sex	Female: 52% (42%), male 48% (58%)
Foreign educated	Medicine/approbation: EU 4%, non-EU 1%
	Rheumatology: EU 1%, non-EU 2%

Source: authors’ own calculation; figures for all rheumatologists (*N* = 1076) excluding residents, based on Kuhlmann et al. (2021)

Only major categories are shown; thus, percentages may not always sum up to 100%

The results presented below reflect the work situation in early 2021 during the end of the second wave of the COVID-19 pandemic in Germany.

### The organization of work

The participants in our survey worked on average 49.6 h per week (women 46.9; men 52.7), with a total range between 20 and 80 h. Women worked on average 5.8 h less per week than their male colleagues, but only 15% worked less than 40 h per week (9% men). The desired worktime was much lower for both men and women, but on average still in the range of fulltime employment (37.9 h per week).

In relation to the desired work hours, the gender gap remained strong but was smaller than for the actual worktime; 3.7 h less in the female compared to the male group. However, the worktime preferences of women and men did not fit into a dichotomous gender pattern of parttime for women, fulltime for men. More than half of the female rheumatologists (52%) preferred fulltime employment of 40 h or higher, while 30% of the male respondents stated less than 40 h per week as their ideal worktime.

The youngest group (under 40 years) worked on average 47.1 h per week, thus 5.1 h less than rheumatologists aged 40 to 50 and 4.1 h less than the group over 50 years. Interestingly, all age groups stated on average an identical desired worktime of 37.9 h per week, which is exactly in the range of fulltime employment.

Furthermore, about two thirds of the respondents (67%) had taken efforts to delegate tasks to other occupational groups, primarily to medical assistants (42%) and RFAs (44%), and to a lesser degree to registered nurses (18%). About 23% were participating in delegation pilot projects and another 6% were planning to do so. All pilot projects focused on RFAs; other occupational groups were marginally represented, such as physician assistants (1%) and non-healthcare workers, e.g., administrative staff (2%).

### Attitudes towards delegation and cooperation

**Fig. 1:**
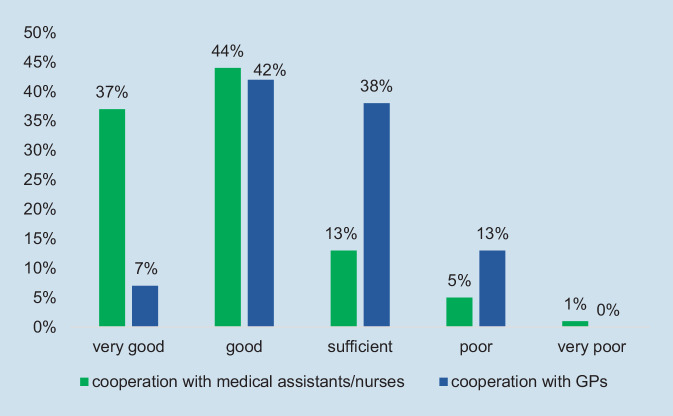
Cooperation with medical assistants/nurses and GPs (general practitioner), perceptions of rheumatologists. (Source: authors’ own figure, based on items 4.1 and 4.2 of the questionnaire. Items: How do you perceive cooperation with GPs/with medical assistants and/or nurses?)

Participants were asked to rate their cooperation on a five-point Likert scale from very good to very poor. The vast majority (81%) perceived their cooperation with medical assistants and/or nurses as good or very good, while cooperation with GPs scored significantly lower, as Fig. 1 illustrates.

**Fig. 2:**
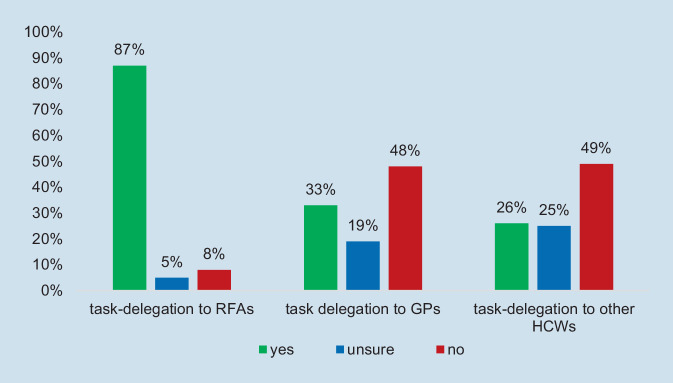
Task delegation as a useful strategy to respond to shortage of rheumatologists, ratings. (Source: authors’ own figure, based on items 4.3, 4.5, 4.7. Items: Do you perceive task delegation to [specialized] RFAs/GPs [after further education]/other healthcare workers as a helpful strategy to respond to shortage of rheumatologists?). *GP* general practitioner, *RFA* “Rheumatologische Fachassistenz”, *HCW* healthcare workers

The vast majority of respondents (87%) agreed that task delegation to specialized RFAs is a helpful strategy to mitigate the shortage of rheumatologists, and most (71%) believed that this can be done without any loss in quality of care if training was obtained in the Curriculum Specialized Rheumatology Assistance (RFA). In contrast, only one third of the respondents (33%) agreed that task delegation to GPs is useful. There was strong concern about the quality of care even if the GPs had obtained further education; only 22% believed there would be no loss of quality. Delegation to other healthcare workers (HCWs) was rated similarly to delegation to GPs, and only slightly more negatively. Figure 2 provides a comparative picture of the attitudes related to task shifting to the three groups.

**Fig. 3:**
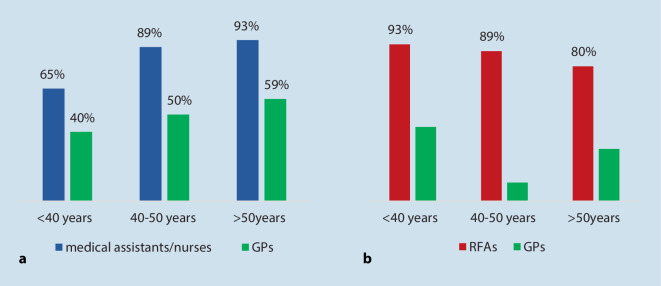
Cooperation with medical assistants/nurses and GPs rated “good” and delegation to RFAs and GPs rated as a useful strategy to respond to shortage of rheumatologists, age groups compared. (Source: authors’ own figure, based on items 4.1 to 4.4. Items: How do you rate cooperation with medical assistants and nurses/with GPs? [Three-point Likert scale]. Do you perceive task delegation to [specialized] RFAs/GPs [after further education] as a helpful strategy to respond to shortage of rheumatologists? [Responses = yes])

No gender-specific pattern could be identified regarding delegation and cooperation, but an interesting age-related pattern emerged: younger age was a predictor for more positive attitudes towards task delegation to both RFAs and GPs, while an adverse age-related effect occurred regarding cooperation with RFAs and with GPs. Figure 3 illustrates the different trends by creating three age groups (<40 years, 40–50 years, >50 years) and four new variables: delegation to RFAs (item 4.3, response yes); delegation to GPs (item 4.4, response yes); cooperation with RFAs (item 4.1, responses very good and good); and cooperation with GPs (item 4.2, responses very good and good).

In the youngest group of respondents, 65% rated cooperation with medical assistants/nurses as good (40% with GPs), compared to higher ratings in the group 50 years and older. As for task delegation, agreement was highest in the youngest group and lowest in the oldest group. The 40 to 50 years age group ranged in between for three of the four items.

Most rheumatologists (74%) were negative or unsure about delegating tasks to other healthcare workers, but the free-text responses also bring new professional groups into view that may be suitable for task delegation. Five groups were mentioned in the responses: physician assistants, orthopedics, other internal medical specialists (e.g., cardiologists, nephrologists), nurses, and social workers/community health workers.

Participants were asked to provide further information and suggestions related to cooperation and task delegation. In contrast to overall positive ratings, the qualitative responses focused on the obstacles to delegation. Participants mentioned legal conditions and lack of financial incentives/remuneration schemes, lack of competences, shortage of medical assistants, complexity of rheumatology tasks, and the need to keep control in the hands of rheumatologists. Few positive comments highlighted opportunities to mitigate shortages through digitalization, changes in the organization of work, and improved interprofessional collaboration and physician networks.

### Work-related stress and burnout

**Fig. 4:**
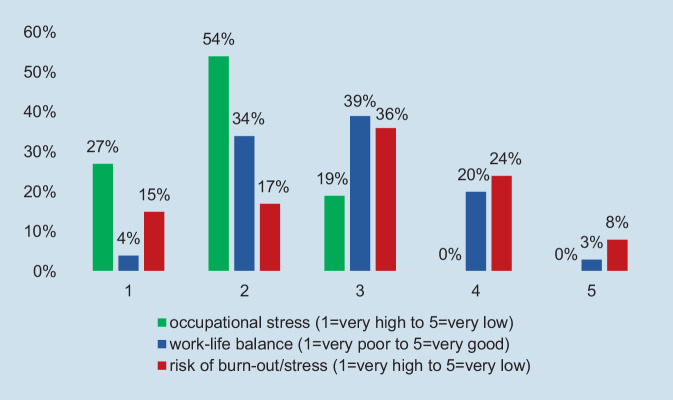
Occupational stress, work–life balance, and risk of developing stress and burnout syndromes, ratings of rheumatologists. (Source: authors’ own figure, based on items 5.1, 5.2, and 5.4. Items: How do you rate your occupational stress/your work-life balance/your current risk of developing stress and burnout syndromes?)

Most respondents rated their own occupational stress as high (54%) or very high (27%) and only few (19) as adequate. A high level of occupational stress might worsen the work–life balance, which was reflected in overall poor ratings (Fig. 4). About one third of the participants rated their current risk of developing stress and burnout syndromes as high (24%) and very high (8%), 36% as moderate, and only about one third as low (17%) or very low (15%).

Figure 4 provides a comparative illustration of the ratings of occupational stress, work–life balance, and mental health risks (stress and burnout syndromes). Some relationship between high levels of occupational stress, poor work–life balance, and risk of burnout seem to be relevant, but the descriptive approach generally does not allow for drawing reliable conclusions on causality. However, the comparative figure (Fig. 4) shows that low levels of stress coincide with an absence of burnout, while the impact of a good work–life balance is not clear.

Of the respondents, 15% reported that they had suffered from burnout and stress syndromes at least once in the past. Consequently, about every sixth rheumatologist has experienced mental health problems. Existing support services for a better work–life balance were rated as poor (42%) and very poor (25%) by about two thirds of the participants; 23% perceived the available support services as adequate and 10% as good or very good. The ratings of support services for coping with occupational stress showed a similar pattern.

Women and men rated work-related stress and their work–life balance largely similarly, yet female respondents ranked available support services for a better work–life balance and for coping with occupational stress more often than men as poor. Also, more women than men perceived themselves to be at risk of stress and burnout; 35% of the female respondents rated their risk as high and very high compared to 29% of the male sample. The number of those suffering from stress and burnout was also slightly higher in the group of women (17.3%) compared to men (13.5%). However, the gender analysis must be interpreted with caution due to the small group size, and this similarly applies to the age-related differences. No coherent age-related trend could be identified. Younger rheumatologists were more likely to rate their risk of stress/burnout syndromes as high and very high than older ones (<40 years = 40%; 40–50 years = 22%; >50 years = 28%). Yet the percentage of those suffering from mental health problems was highest among rheumatologists aged 40 to 50 years (33% of the age group, compared to <40 years = 12%; >50 years = 10%).

### Discrimination and sexual violence

About half of the participants had experienced gender discrimination in their everyday work life, 17% frequently and 32% seldom. Other forms of discrimination (e.g., related to ethnicity) were less frequent, but still more than every fifth respondent had experienced these forms (15% seldom, 6% frequently). We cannot specify the reasons for discrimination, but migration does not fully explain the findings. To recall, only 5% of the sample did not obtain their medical approbation in Germany and only 3% were foreign-trained in rheumatology (Table 1).

Participants were also asked to rate gender discrimination and other forms of discrimination in relation to their career development. Fewer respondents had experienced career discrimination compared to general discrimination experiences (4% for gender, 8% for other discrimination). Furthermore, every fourth rheumatologist stated that they had experienced sexual harassment/violence in their everyday work life, 23% seldom and 2% frequently, and even more have observed this (35% seldom, 2% frequently).

**Fig. 5:**
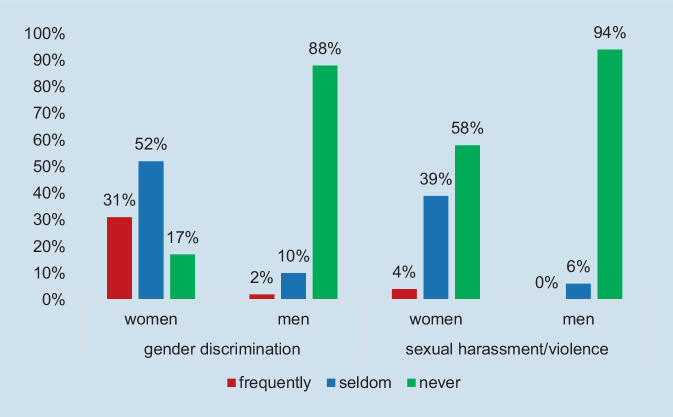
Gender discrimination and sexual harassment/violence by sex category. (Source: authors’ own figure, based on items 7.1, 7.5. Items: Have you experienced gender discrimination/sexual harassment or violence in your everyday work-life?)

Women experienced all forms of discrimination much more often than men (Fig. 5). 83% of the female respondents reported gender discrimination (31% frequently) and 43% sexual harassment or violence (4% frequently). In the male group, 13% of the respondents had experienced some form of gender discrimination (2% frequently) and 6% sexual harassment/violence (nobody frequently). As for age group differences, younger respondents were more often affected by gender discrimination, including career-related discrimination, than older ones, yet no coherent age-related pattern could be identified for the other discrimination items.

### The impact of COVID-19 on the work situation

The COVID-19 pandemic has affected all healthcare workers at least to some degree; infection control has been strengthened in all areas. The actual impact of the pandemic is context dependent and varies significantly, however. About two thirds of the respondents stated a strong (50%) or very strong (14%) increase in occupational stress, 27% perceived no changes, and 9% a reduction in stress. Only few respondents reported changes in the delegation and coordination of tasks; 12% introduced new forms of task delegation and an equally low percentage perceived changes in the cooperation with GPs (6% to the better and 6% to the worse). Additional qualitative responses (free text) highlighted an increase in task delegation to RFAs, including administrative as well as patient-related tasks, and improved collaboration during the pandemic.

About half of the rheumatologists in the survey (49%) stated that the pandemic has led to radical changes at their workplace. The vast majority provided further qualitative information. Overall, 44 free-text responses give proof of the high relevance of this topic. Four major categories of workplace changes during the pandemic emerged from the material: workload (qualitative and quantitative dimensions), work life, organization of work, and service delivery. Workload and work life refer to changes at the individual level, while the other two categories reflect changes at the organizational level of work. Table 2 below provides examples for each of the four categories.

**Table 2:** Radical changes at the workplace caused by the COVID-19 pandemic

Category	Examples of changes
Workload, new tasks and demands (individual level)	Higher workload through hygiene measures and increased responsibility
	High burden through an increase in phone calls and digital services
	Many deaths
	Very high demand for communication
	Patient education on vaccination and behavioral issues; patients demanding information on vaccination and therapies related to the pandemic
	Continuing change, new demands, and lack of work routine
	Increased demand of other units for COVID-19-related information (immune suppression)
	Strong increase in digitalization, missing competences of the population
	Patient education on digital services
	Expanded communication with patients to mitigate COVID-19 restrictions and prohibited visits of relatives
Work life (individual level)	Less time due to higher individual workload related to childcare
	Risk of infection; social isolation
	Wearing face masks is exhausting, social distancing not always possible
	Part-time work because of childcare responsibility
	Relocation to a COVID-19 ward with alternating shift duty
	Relocation to another specialty due to unit closing
	Missing academic conferences
	Weekend shifts due to changes in hospital admission
Organization of work (organizational level)	Increase of email, phone, and online counselling, consequently less predictable organization, no clear structure of office hours
	Home office
	Online/video-based office hours, phone calls
	Increase in work hours, often caused by strong increases in digital services
	Increase in demand for patient education and information
	Tasks taken over from GPs, higher demand for patient education
	Changes in the organization of the unit/the surgery, changing time management
	Patients increasingly cancel appointments without notice
	Improved patient management through better planning
Service delivery (organizational level)	Financial losses
	More time required per patient
	Extended length of stay of COVID-19-positive patients
	Reduced number of beds, shortened length of stay of patients, admissions during weekend
	Investigation in personal protective equipment (PPE) and new equipment to improve infection control and hygiene
	Poor quality of care of non-COVID-19 patients
	Reduced quality of care due to strong increase in patient phone calls
	Reduced number of beds, reduced number of patients
	Social distancing rules and hygiene measures reduce the number of patients permitted to be in the surgery
	Longer waiting lists of patients

Source: authors’ own table, based on item 6.4, qualitative information; responses translated (verbatim and paraphrased)

Item: Has the pandemic led to any radical changes at your workplace (next to hygiene measures)? If yes, which ones?

The qualitative findings illustrate that the pandemic did not only cause more work and a higher workload. Moreover, COVID-19 affected the work situation of rheumatologists in many different ways and at various levels, as the examples in Table 2 highlight. The respondents reported strong increases in workload and stress caused by changes in the individual work situation (e.g., increased workload in childcare) as well as by organizational changes (Table 2, organization of work). Digitalization appeared to be a cross-cutting category and a strong driver of high workload and stress, e.g., through increased online counselling, video-based office hours, or missing competences of the population. A significant increase in the communication needs of patients and a new demand for patient education, especially on vaccination and on therapies and behavior related to COVID-19, were reported as further stressors (Table 2, workload/new tasks).

## Discussion and perspectives

In this article, we introduced a health workforce approach and argued that the work situation of rheumatologists provides important policy levers to improve recruitment and retention, especially under conditions of growing shortage and limited opportunities for competition. The findings have illustrated the usefulness of this approach. Major policy levers emerging from the research are specified below.

### Delegation and cooperation: opportunities and challenges

Positive attitudes towards task delegation and cooperation with medical assistants, in particular with RFAs, highlighted opportunities to mitigate shortages through innovation in the organization of work. The findings point in a similar direction as the literature [13, 15] but also bring task delegation to other healthcare workers into view. Besides new opportunities, the data uncovered barriers to innovation in interprofessional cooperation, an area that has received little attention in the literature so far. Overall poor ratings and strong concern about the quality of care if tasks were to be delegated to GPs indicated that the implementation of new forms of collaboration may be challenging. Age-related trends were especially alarming, as younger rheumatologists more often expressed negative attitudes towards collaboration with GPs than older respondents. The literature showed that rheumatologists’ negative views on GPs’ capability to take over tasks from rheumatologists were echoed by GPs’ perceptions of the cooperation as poor [36].

Negative attitudes towards cooperation may hamper effective interprofessional cooperation and turn individual perceptions into a health policy issue for two reasons: cooperation between the two groups of physicians is essential to provide person-centered and integrated care—the health services argument. Cooperation is also a cornerstone of a future rheumatology workforce, which uses the skills of diverse professional groups most effectively—the health workforce argument.

### Worktime preferences: changing age- and gender-related patterns

Our survey confirmed long work hours and overtime work as the rule rather than the exception in rheumatology, which mirrors a general problem of German physicians. Working hours are generally above legally defined fulltime employment in Germany (38 to 42 h, depending on the sector) [34]. From a health workforce planning perspective, long work hours might appear to mitigate shortages and increase workforce capacity. However, this raises concerns about physicians’ health and wellbeing [34] as well as about the sustainability and resilience of workforce arrangements. Trends in the younger age group pointed towards a preference for slightly shorter work hours. This trend may be reinforced in the future by an increase in female physicians, as observed in all areas of medicine [22].

Common assumptions that women prefer parttime work were not supported. Women had on average fewer work hours than men, but still worked on average 47 h per week, which is far above fulltime employment. The findings suggest that we should neither assume that all women are lingering for a parttime position, nor that all men wish to be fulltime physicians. Future health workforce planning in rheumatology should consider that worktime preferences of women and men are more diverse than existing gender stereotypes and that both women and men aim for shorter work hours. To recall, a good work–life balance was identified as one important factor that can make rheumatology attractive for resident physicians [6].

### Gender discrimination and sexual violence: the hidden risks of work life

The research made discrimination and sexual violence (including harassment) in rheumatology visible. Female sex was a very strong predictor for all items: awareness and experience of discrimination, as well as harassment and violence. The results are supported by the literature. In the EULAR survey [30], a high proportion of respondents reported having experienced gender discrimination (42%; in our survey 49%) and sexual harassment/violence (26%; in our survey 25%). Women reported having experienced sexual harassment/violence nearly three times more often than men (3.6 times in our survey). Discrimination and violence most often threaten the mental health of female rheumatologists but may also affect men [1, 16, 30]. Discrimination may take many different forms and is not limited to gender. It is important to understand that all forms of discrimination and sexual harassment/violence are very serious workplace stressors [25], threatening the health of individual physicians and hampering or even damaging the careers of many women and a few men.

While public attention to gender equality and sensitivity to discrimination and harassment has increased over recent years at least to some degree, especially through equal opportunity laws and policies and the #MeToo movement, scholarly debate in rheumatology largely ignores the problems, and this also applies to health policy (the EULAR survey has been discussed previously as an important exception [30]). There is an urgent need to take action, as discrimination and harassment experiences provoke dropouts and may exacerbate existing workforce shortages, next to the individual mental health risks and career obstacles.

### Workplace stress and mental health risks: COVID-19 as an amplifier

The findings reveal that every sixth rheumatologist has suffered from stress at least once in the past and the poor ratings of support services are a wakeup call. High levels of stress and mental health risks were also reported by other studies, although the results from different countries and surveys (which have applied different research aims and use different methods) may not be fully comparable [25, 28, 38]. Our material revealed that COVID-19 was an amplifier of stress. Not all rheumatologists stated higher workload during the pandemic and not everybody may be at risk of developing burnout syndromes. Yet the widespread perception among rheumatologists of increased stress and lack of control at the workplace during the pandemic, together with new demand for services and increased pressures from patients, highlight the severity of the problem. The findings also indicate that infection prevention and vaccination prioritization are important but not sufficient. Mental health programs and targeted social support services for rheumatologists (and other healthcare workers) must become a priority issue of health policy and pandemic preparedness [10, 40].

Digitalization and an increased demand for communication appeared to be relevant single stressors of increased stress und workload during the COVID-19 pandemic. Yet the major problem might be the magnitude of changes and new demands, together with a lack of routine to respond to the challenges. The radical changes caused by COVID-19 require further investigation to better understand the perceptions and needs of rheumatologists and to respond effectively to the changing work situation. This may help improve individual work life and ultimately the resilience of the workforce.

### Limitations

Using an online survey made it possible to collect data during the pandemic in a short time and with limited resources. The study has relevant limitations, however. Our focus on the workplace and the individual perceptions and the use of descriptive methodology provided a helpful overview of the health workforce situation in rheumatology, but it should be considered as explorative and did not aim to identify or verify causal statistical relationships. Furthermore, we were unable to calculate a reliable response rate because we did not know how many rheumatologists actually received and read the social media invitations. Younger rheumatologists and women were overrepresented, reflecting common trends of higher use of digital services in younger groups and the greater willingness of women to respond to surveys. However, an accurate comparison was not possible, because available public statistics only provide the number of practicing rheumatologists and lack information on the number of residents.

This descriptive approach highlighted both opportunity for innovation and problematic work experiences but did not allow us to identify the institutional conditions or to explore more specific intervention strategies; the sample size further reduced the opportunities for subgroup analyses. The free-text responses offered some opportunity for qualitative analysis and provided in-depth information on the individual work situation, yet the material was limited and did not include information on context. The research helped identify relevant topics to be explored in future research, including qualitative investigations.

## Summary recommendations

- Sustainable workforce management must take changing worktime preferences into account and prevent extensive overtime work to create healthy work conditions that may improve retention and attract more physicians to rheumatology.
- Positive perceptions of collaboration and task delegation to subordinated healthcare workers may help mitigate shortages, but the inclusion of other healthcare workers, improved collaboration with GPs, and effective policy incentives are needed.
- Strengthening gender equality and preventing discrimination and sexual harassment and violence in rheumatology must become a management and leadership goal to prevent career damages, stress, and job leaves.
- COVID-19 is an amplifier of stress, with digital services, an increased demand for communication, and new needs for patient education being relevant stressors.
- Building back better after the pandemic must include efforts to reduce workplace stress and improve mental health in the rheumatology workforce

## Supplementary Information

Survey questionnaire

## Declarations

Approval by the Ethics Committee of Medical School Hannover was obtained for the online survey; Nr 9441_BO_K_2020.
