# Supplementary material for: Work situation of rheumatologists and residents in times of COVID-19: Findings from a survey in Germany
Source: Z Rheumatol. 2021 Sep 17;82(4):331–41. doi: 10.1007/s00393-021-01081-5 (PMC8448391; doi:10.1007/s00393-021-01081-5)
Supplement: Supplementary file 1 — Survey questionnaire [file 393_2021_1081_MOESM1_ESM.pdf]

MHH Medizinische Hochschule Hannover

Care4Rheumatology → Care4Rheumatology 05.07.2021, 09:59

Seite 01

Care4Rheumatology: Fachkräftesituation, Arbeits- und Ausbildungsbedingungen in der Rheumatologie in Deutschland

Die Studie möchte einen fundierten Beitrag zur Verringerung des **Fachkräftemangels in der Rheumatologie** und zur Verbesserung der Arbeits- und Ausbildungssituation leisten. Bisher mangelt es an systematischen Informationen, deshalb sind Ihre Erfahrungen und Einschätzungen sowie Vorschläge zur Verbesserung entscheidend! Die Befragung ist anonym und wird deutschlandweit mit internistischen Rheumatologinnen und Rheumatologen und denjenigen in Weiterbildung online mittels eines standardisierten Fragebogens durchgeführt.

Im Fokus stehen die **Arbeits- und Ausbildungsbedingungen**. Die Befragung dauert etwa 5 Minuten. Die Studie wird an der Medizinischen Hochschule Hannover (MHH), Klinik für Rheumatologie und Immunologie durchgeführt und von der Deutschen Gesellschaft für Rheumatologie e.V. (DGRh) unterstützt. Als Ansprechpartnerin und für Fragen steht Ellen Kuhlmann (Dr. habil., MPH, kuhlmann.ellen@mh-hannover.de) zur Verfügung.

Einwilligungserklärung und Informationen zum Datenschutz

Ich bin über die Ziele der Befragung mit einem Informationsblatt (Projektflyer) informiert worden. Ich erkläre mich bereit, an der Befragung teilzunehmen und bin damit einverstanden, dass meine Antworten anonym ausgewertet und als Material für wissenschaftliche Zwecke genutzt werden können. Der Link zu der Befragung (https://webext.mh-hannover.de/soscisurvey/Care4Rheumatology/) wird durch die MHH und die Deutsche Gesellschaft für Rheumatologie e.V. (DGRh) und weitere berufliche/fachspezifische Interessenvertretungen zur Verfügung gestellt. Der Link erfolgt über das Portal SoSci, das auf einem MHH Server gehostet wird und keine IP Adresse erfasst. Mir ist bekannt, dass ich die Teilnahme während des Ausfüllens jederzeit ohne Angabe von Gründen abbrechen kann, ohne dass mir hieraus Nachteile entstehen könnten. Nach Absenden des Fragebogens ist eine Rücknahme nicht möglich und weitere Rechte nach DSGVO sind nicht umsetzbar, da eine Identifizierung der einzelnen Teilnehmenden nicht möglich ist. Bei Freitextangaben bitte keine personenbeziehbaren oder personenbezogenen Angaben machen, um die Anonymität zu sichern.

- ◯ Ja
- ◯ Nein

Seite 02

Ohne Ihre Einwilligung ist es uns leider nicht möglich, ihre Antworten in der Auswertung zu berücksichtigen.
Wir bitten um die Einwilligung.

Seite 03

1 Allgemeine Angaben zur beruflichen Tätigkeit

1.1 Ärztlich tätig als

- ◯ Internistische Rheumatologin/ Rheumatologe
- ◯ Internistische Rheumatologin/ Rheumatologe mit Weiterbildungsermächtigung
- ◯ Weiterbildungsassistentin/ Weiterbildungsassistent
- ◯ andere

1.2 Tätigkeitsbereich (Mehrfachnennungen möglich)

- ☐ Stationär
- ☐ In leitender Funktion
- ☐ Ambulant
- ☐ Niedergelassen
- ☐ Einzelpraxis
- ☐ Gemeinschaftspraxis, etc.
- ☐ Angestellt
- ☐ Andere, welche? ______

1.3 In welchem Bundesland liegt das Krankenhaus/ die Praxis?

- ◯ Altes Bundesland
- ◯ Neues Bundesland

In welchem Gebiet liegt das Krankenhaus/ die Praxis?

- ◯ Großstadt, >100.000 Einwohner
- ◯ Mittelstadt, 20.000-100.000 Einwohner
- ◯ Kleinstadt, 5.000-20.000 Einwohner
- ◯ Landgemeinde, <5000 Einwohner

2 sozio-demographische Angaben

2.1 Geschlecht

- ◯ weiblich
- ◯ männlich
- ◯ divers

2.2 Medizinische Ausbildung und Approbation in

- ◯ Deutschland
- ◯ EU (ohne Deutschland)
- ◯ nicht-EU Ausland

2.3 Weiterbildung zum Facharzt/ Fachärztin Rheumatologie

- ◯ Deutschland
- ◯ EU (ohne Deutschland)
- ◯ nicht-EU Ausland

2.4 Altersgruppe

- ◯ < 35 Jahre
- ◯ 35-40 Jahre
- ◯ 40-50 Jahre
- ◯ 50-60 Jahre
- ◯ 60-<66 Jahre
- ◯ ≥66 Jahre

Seite 05

3 Arbeitszeit und Arbeitsorganisation

3.1 Wöchentliche durchschnittliche Arbeitszeit

[ ]

3.2 Wunscharbeitszeit pro Woche

[ ]

3.3 Wurden in Ihrem Arbeitsbereich Maßnahmen der Delegation ärztlicher Aufgaben an andere Berufsgruppen eingeführt?

- ○ ja
- ○ nein

Wenn ja:

- ☐ Delegation an MFAs
- ☐ Delegation an RFAs
- ☐ Delegation an Gesundheits- und Krankenpflegerinnen/-pfleger
- ☐ Delegation an andere Berufsgruppen? Welche? [ ]

3.4 Nehmen Sie bzw. Ihr Arbeitsbereich an Pilotprojekten zu Delegation (z.B. Rheumatologische Fachassistenz, RFAs) und/oder zu neuen Berufsgruppen teilt?

- ○ ja
- ○ nein
- ○ in Planung

Wenn ja, welche Berufsgruppen sind einbezogen?

- ☐ RFAs
- ☐ Physician Assistants
- ☐ andere, welche? [ ]

4 Einschätzungen zu Kooperation und Aufgabendelegation

4.1 Wie schätzen Sie Ihre Kooperation mit Hausärztinnen und Hausärzten ein?

sehr gut	gut	ausreichend	schlecht	sehr schlecht
**4**	**3**	**2**	**1**	**0**
○	○	○	○	○

4.2 Wie schätzen Sie Ihre Kooperation mit den Medizinischen Fachangestellten und/oder den Pflegekräften ein?

sehr gut	gut	ausreichend	schlecht	sehr schlecht
**4**	**3**	**2**	**1**	**0**
○	○	○	○	○

4.3 Halten Sie eine Aufgabendelegation an (speziell ausgebildete) Rheumatologische Fachassistenz (RFAs) für sinnvoll, um auf den Fachkräftemangel in der Rheumatologie zu reagieren?

○ ja
○ nein
○ ich bin unsicher

4.4 Denken Sie, dass eine Aufgabendelegation zukünftig ohne Qualitätsverlust für die Versorgung möglich wäre, wenn eine entsprechende Qualifikation im Rahmen des Curriculums Rheumatologische Fachassistenz vorliegt?

○ ja
○ nein
○ ich bin unsicher

4.5 Halten Sie eine Aufgabendelegation an Hausärztinnen und Hausärzte (nach entsprechender Fortbildung) für sinnvoll, um auf den Fachkräftemangel zu reagieren?

○ ja
○ nein
○ ich bin unsicher

4.6 Denken Sie, dass eine Aufgabendelegation zukünftig ohne Qualitätsverlust für die Versorgung möglich wäre, wenn eine entsprechende Fortbildung von Hausärztinnen und Hausärzten erfolgt?

○ ja
○ nein
○ ich bin unsicher

4.7 Halten Sie eine Aufgabendelegation an andere Berufsgruppen für sinnvoll?

- ○ ja, welche? [ ]
- ○ nein
- ○ ich bin unsicher

4.8 Haben Sie weitere Kommentare oder Anregungen zu Kooperation und Aufgabendelegation?

[ ]

5 Einschätzungen zur eigenen Arbeitssituation

5.1 Wie schätzen Sie Ihre eigene berufliche Arbeitsbelastung ein?

sehr hoch	hoch	angemessen	niedrig	sehr niedrig
○	○	○	○	○

5.2 Wie schätzen Sie Ihre Work-life Balance ein?

sehr gut	gut	ausreichend	schlecht	sehr schlecht
○	○	○	○	○

5.3 Wie schätzen sie die vorhandenen Unterstützungsangebote zur Verbesserung der Work-life Balance in ihrem Arbeitsbereich ein?

sehr gut	gut	ausreichend	schlecht	sehr schlecht
○	○	○	○	○

5.4 Wie schätzen Sie aktuell Ihr Risiko ein, an Stress oder Burn-out Syndromen zu erkranken?

sehr hoch	hoch	mäßig	gering	sehr gering
○	○	○	○	○

5.5 Waren Sie in der Vergangenheit bereits einmal an Burn-out Syndromen erkrankt?

- ○ ja
- ○ nein

5.6 Wie schätzen Sie die Unterstützungsangebote in ihrem Arbeitsbereich zur Bewältigung von Stress und beruflichen Belastungen ein?

sehr gut	gut	ausreichend	schlecht	sehr schlecht
○	○	○	○	○

6 COVID-19 Pandemie: Auswirkungen im eigenen Arbeitsbereich

6.1 Haben sich Ihre beruflichen Belastungen verändert?

stark erhöht	erhöht	unverändert	verringert	stark verringert
○	○	○	○	○

6.2 Wurden neue Formen von Aufgabendelegation eingeführt?

○ ja, welche?

○ nein

6.3 Hat sich die Kooperation mit Hausärztinnen und Hausärzten verändert?

stark verbessert	verbessert	unverändert	verschlechtert	stark verschlechtert
○	○	○	○	○

6.4 Hat die Pandemie (neben den Hygienemaßnahmen) zu einschneidenden Veränderungen in ihrem Arbeitsbereich geführt?

○ ja, welche?

○ nein

7 Diskriminierungserfahrungen *(angelehnt an EULAR Befragung 2019)*

7.1 Haben Sie selbst aufgrund ihres Geschlechts Diskriminierung im beruflichen Alltag erfahren?

- ◯ häufiger
- ◯ selten
- ◯ nie

7.2 Haben Sie selbst andere Formen der Diskriminierung (z.B. ethnische Zugehörigkeit) im beruflichen Alltag erfahren?

- ◯ häufiger
- ◯ selten
- ◯ nie

7.3 Haben Sie selbst aufgrund ihres Geschlechts Diskriminierung in ihrer Karriereentwicklung erfahren?

- ◯ häufiger
- ◯ selten
- ◯ nie

7.4 Haben Sie selbst andere Formen der Diskriminierung (z.B. ethnische Zugehörigkeit) in ihrer Karriereentwicklung erfahren?

- ◯ häufiger
- ◯ selten
- ◯ nie

7.5 Haben Sie selbst sexuelle Belästigung oder Gewalt im beruflichen Alltag erfahren?

- ◯ häufiger
- ◯ selten
- ◯ nie

7.6 Haben Sie in Ihrem beruflichen Alltag sexuelle Belästigung oder Gewalt beobachtet oder wissen davon?

- ◯ häufiger
- ◯ selten
- ◯ nie

Seite 10
8WE

8 Weiterbildungsermächtigte

8.1 Wie viele Weiterbildungsassistentinnen/-assistenten beschäftigen Sie gegenwärtig?

[ ]

8.2 Wie viele Weiterbildungsassistentinnen/-assistenten würden Sie gern beschäftigen?

[ ]

8.3 Wie bewerten Sie aktuell den Arbeitsmarkt für Weiterbildungsassistentinnen/-assistenten in der Rheumatologie?

- ○ Freie Stelle können nicht besetzt werden aufgrund fehlender Bewerbungen
- ○ Es gibt hinreichend qualifizierte Bewerberinnen/ Bewerber

8.4 Haben Sie Vorschläge zur Verbesserung der Weiterbildung?

- ○ ja [ ]
- ○ nein

Seite 11
9WA

9 Weiterbildungsassistentinnen und -assistenten

9.1 Wie bewerten Sie aktuell den Arbeitsmarkt für Weiterbildungsassistentinnen/-assistenten in der Rheumatologie aufgrund Ihrer Erfahrungen?

- ◯ Es gibt hinreichend freie Stellen.
- ◯ Freie Stellen konzentrieren sich auf bestimmte Regionen und Kliniken, welche? ____
- ◯ Ich hatte Probleme, eine Weiterbildungsstelle zu finden.

9.2 Haben Sie die Weiterbildungsstelle erhalten, die Sie angestrebt haben?

- ◯ ja
- ◯ nein
- ◯ ich bin mir noch unsicher

9.3 Wie schätzen Sie die Qualität Ihrer Weiterbildung ein?

sehr gut	gut	ausreichend	schlecht	sehr schlecht
◯	◯	◯	◯	◯

9.4 Wie bewerten Sie aktuell Ihre eigene Betreuungssituation in der Weiterbildung?

Wie bewerten Sie aktuell Ihre eigene Betreuungssituation in der Weiterbildung?

sehr gut	gut	ausreichend	schlecht	sehr schlecht
◯	◯	◯	◯	◯

9.5 Wie schätzen Sie Ihre Karrierechancen ein?

Wie schätzen Sie Ihre Karrierechancen ein?

sehr gut	gut	ausreichend	schlecht	sehr schlecht
◯	◯	◯	◯	◯

9.6 Haben Sie bereits berufliche Pläne nach Abschluss Ihrer Weiterbildung?

- ◯ Tätigkeit im Krankenhaus
- ◯ Niederlassung
- ◯ angestellt in Praxis
- ◯ akademische Laufbahn
- ◯ Ausland
- ◯ andere, welche? ________
- ◯ keine Pläne

9.7 Haben Sie Vorschläge zur Verbesserung der Weiterbildung?

- ◯ ja, welche? ________
- ◯ nein

Letzte Seite

Vielen Dank für Ihre Teilnahme!

Wir möchten uns ganz herzlich für Ihre Mithilfe bedanken.

Ihre Antworten wurden gespeichert, Sie können das Browser-Fenster nun schließen.

Ellen Kuhlmann – 2021
